# Identification of disulfidptosis-related subgroups and prognostic signatures in lung adenocarcinoma using machine learning and experimental validation

**DOI:** 10.3389/fimmu.2023.1233260

**Published:** 2023-09-20

**Authors:** Yuzhi Wang, Yunfei Xu, Chunyang Liu, Chengliang Yuan, Yi Zhang

**Affiliations:** ^1^ Department of Laboratory Medicine, Deyang People’s Hospital, Deyang, Sichuan, China; ^2^ Department of Laboratory Medicine, Chengdu Women’s and Children’s Central Hospital, Chengdu, Sichuan, China; ^3^ Department of Ultrasound, The First People’s Hospital of Yibin, Yibin, Sichuan, China; ^4^ Department of Blood Transfusion, Deyang People’s Hospital, Deyang, Sichuan, China

**Keywords:** disulfidptosis, lung adenocarcinoma, prognosis, tumor microenvironment, immunotherapy

## Abstract

**Background:**

Disulfidptosis is a newly identified variant of cell death characterized by disulfide accumulation, which is independent of ATP depletion. Accordingly, the latent influence of disulfidptosis on the prognosis of lung adenocarcinoma (LUAD) patients and the progression of tumors remains poorly understood.

**Methods:**

We conducted a multifaceted analysis of the transcriptional and genetic modifications in disulfidptosis regulators (DRs) specific to LUAD, followed by an evaluation of their expression configurations to define DR clusters. Harnessing the differentially expressed genes (DEGs) identified from these clusters, we formulated an optimal predictive model by amalgamating 10 distinct machine learning algorithms across 101 unique combinations to compute the disulfidptosis score (DS). Patients were subsequently stratified into high and low DS cohorts based on median DS values. We then performed an exhaustive comparison between these cohorts, focusing on somatic mutations, clinical attributes, tumor microenvironment, and treatment responsiveness. Finally, we empirically validated the biological implications of a critical gene, KYNU, through assays in LUAD cell lines.

**Results:**

We identified two DR clusters and there were great differences in overall survival (OS) and tumor microenvironment. We selected the "Least Absolute Shrinkage and Selection Operator (LASSO) + Random Survival Forest (RFS)" algorithm to develop a DS based on the average C-index across different cohorts. Our model effectively stratified LUAD patients into high- and low-DS subgroups, with this latter demonstrating superior OS, a reduced mutational landscape, enhanced immune status, and increased sensitivity to immunotherapy. Notably, the predictive accuracy of DS outperformed the published LUAD signature and clinical features. Finally, we validated the DS expression using clinical samples and found that inhibiting KYNU suppressed LUAD cells proliferation, invasiveness, and migration in vitro.

**Conclusions:**

The DR-based scoring system that we developed enabled accurate prognostic stratification of LUAD patients and provides important insights into the molecular mechanisms and treatment strategies for LUAD.

## Introduction

Lung cancer is a global health concern and one of the foremost sources of cancer patient morbidity and mortality, posing a grave threat to public health (1). In 2020, an estimated 2,206,771 new cases were diagnosed, and 1,796,144 fatalities occurred (2). Non-small cell lung cancer (NSCLC) stands as the prevalent pathological category among lung cancers, comprising about 85% of all cases, whereas lung adenocarcinoma (LUAD) is the most frequently occurring subtype of NSCLC (3). LUAD is plagued by a high incidence of invasive behavior and metastatic spread. However, significant advances in the treatment of LUAD have been achieved in the past few decades. Accordingly, targeted therapies and immunotherapies have been demonstrated to enhance the treatment efficacy and outcomes for patients with LUAD (4). However, the majority of patients develop drug resistance and relapse following initial treatment, resulting in no significant improvement in 5-year survival rates (5). Thus, to benefit more LUAD patients, there is an urgent need to identify new therapeutic targets and prognostic indicators for predicting survival and guiding clinical treatment in LUAD patients.

Programmed cell death (PCD) is a cellular death process governed by a molecular program that is controlled by particular genes. This process is essential for the normal development of organisms and the preservation of homeostasis (6). Exploration and characterization of these cell death mechanisms not only deepens our fundamental comprehension of cellular equilibrium, but also offers valuable perspectives for the therapeutic approach to a variety of diseases, including cancer. For instance, recent studies have progressively highlighted the tumor-inhibiting effects of ferroptosis, which is achieved through the deprivation of cysteine and the generation of reactive oxygen species (ROS) by p53 (7, 8). Moreover, the enhancement of lipid peroxidation by activated CD8+ T cells can induce ferroptosis, which contributes to the antitumor efficacy of immunotherapy (9). The role of autophagy in cancer can vary, depending on the specific tumor model and tumor stage. During the initial stages of cancer, autophagy functions as a protective mechanism, shielding normal cells from tumorigenesis by preventing DNA damage and mutations (10). However, in the context of fully-formed solid tumors, autophagy shifts its role and promotes tumor progression by promoting tumor growth, enhancing cell survival, enhancing resistance to platinum-based chemotherapy, and facilitating the formation of metastases (11). Autophagy inhibitors, therefore, comprise one of the treatment options for patients with advanced tumors. Recent research (Liu et al., 2023) demonstrates that excessive accumulation of disulfide induces a unique form of controlled cell death known as “disulfidptosis” that is distinct from apoptosis, necrosis, autophagy, and ferroptosis (12). SLC7A11 is an essential transport protein whose primary function is to facilitate the cellular uptake of cysteine (13). Cysteine is a necessary building block for the synthesis of glutathione and a crucial component for inhibiting oxidative stress in cells and regulating iron death pathways (14). However, it has also been shown to possess certain cytotoxic properties (15). Gan Bo et al., discovered that under conditions of glucose deprivation, high expression of SLC7A11 leads to a significant consumption of NADPH within cells, abnormal aggregation of disulfides like cysteine, inducing disulfide stress and rapid cell death (16). This form of cell death induced by glucose deprivation and high SLC7A11 expression in cancer cells cannot be prevented by inhibitors of cell death that act on other cells, nor is it caused by depletion of intracellular ATP. However, thiol-oxidizing agents, such as Diamide, can enhance this effect. Moreover, under glucose-deficient conditions, the number of disulfide bonds in the actin cytoskeleton increases significantly. Therefore, this study suggests that the induction of disulfide-dependent cell death by GLUT inhibitors may be an effective cancer treatment strategy. Consequently, focusing on disulfidptosis regulators (DRs) as potential targets provides new perspectives for understanding the complexities of the occurrence and development mechanism in LUAD. This approach is, therefore, of significant importance in enhancing the efficacy of treatment in patients with LUAD patients. However, the full scope of the impact of DRs on outcomes and treatments for LUAD patients has yet to be comprehensively explored.

In the current study, we divided 1569 LUAD samples into two disulfidptosis-associated subtypes according to 18 DRs and compared survival and immune infiltration between the subtypes. We also developed a disulfidptosis score (DS) to predict overall survival (OS) and to delineate the immunological landscape of LUAD. As indicated by the findings, a higher DS was associated with unfavorable prognostic outcomes and worse immunotherapy responses in LUAD, suggesting the potential clinical utility of DS as a tool for assessing prognosis and immunotherapy efficacy. Thus, the current study introduces an innovative approach for assessing the efficacy of immunotherapy and predicting the prognosis of LUAD patients based on DS.

## Materials and methods

### Data collection

The LUAD data were obtained from The Cancer Genome Atlas (TCGA)-LUAD (https://portal.gdc.cancer.gov/), GSE31210, GSE68465, and GSE72094 in Gene Expression Omnibus (GEO) (https://www.ncbi.nlm.nih.gov/geo/), which included RNA sequencing data, somatic mutation data, copy number variation (CNV) data, and corresponding clinical information. The TCGA-LUAD cohort, which consisted of 539 LUAD tissues and 59 cancer-adjacent tissue samples, served as a training cohort. Meanwhile, GSE31210, GSE68465, and GSE72094 were utilized for the validation cohort, which consisted of 1,150 LUAD patients. Additionally, the “sva” package was used to correct the batch effect between the different datasets by adopting the “combat” algorithm (17). Moreover, patients who lacked OS time were filtered out. Finally, 1569 eligible patients were encompassed in the study. The detailed clinical characteristics of all LUAD patients is listed in **Supplementary Table 1**. Additionally, 18 DRs were collected from the previous study (**Supplementary Table 2**) (12).

### Consensus cluster analysis of DRs

A consensus clustering algorithm was employed to discern optimal subtypes founded on the expression of 18 DRs using the R package “ConsensusClusterPlus”. The number of clusters (K) and their stability (with 1,000 repeats for mast k = 9) were determined by the consensus clustering algorithm (18). The clustering was based on dividing centromeres with “Euclidean” distances (the most common and familiar distance measurement methods and correlation of K-Means clustering). Additionally, a T-distributed Stochastic Neighbor Embedding (tSNE) analysis was conducted to decrease the dimensions and differentiate the subtypes of information (tSNE can preserve local similarities between data points and is one of the most used unsupervised clustering visualization methods).

### Differentially expressed genes and functional annotation

DEGs among DRs subtypes were determined using the “limma” R package with the filtering criteria of log (fold change) >1 and False-discovery rate (FDR) <0.05 (19). To further investigate the potential functions and enriched pathways of DEGs, functional enrichment analyses were conducted on DEGs employing the “clusterprofiler” R package (20).

### Generation of DS

The DS was calculated to quantify the disulfidptosis patterns of the LUAD. Accordingly, a univariate cox regression analysis was conducted to identify the DEGs are related to prognosis. Subsequently, the patients were segregated into distinct gene cluster groups according to the expression of prognostic DEGs using the unsupervised clustering method. Based on prognostic DEGs, the 10 machine learning algorithms, including ‘Least Absolute Shrinkage and Selection Operator (LASSO, “glmnet” package)’, ‘Ridge (“glmnet” package)’, ‘Elastic network (“glmnet” package)’, ‘StepCox (“survival” package)’, ‘Survival support vector machine (survival-SVM, “survivalsvm” package)’, ‘CoxBoost (“CoxBoost” package)’ (21), ‘Supervised principal components, “superpc” package’ (22), ‘partial least squares regression for COX, “plsRcox” package’, ‘random survival forest (RSF, “randomForestSRC” package)’, ‘generalized boosted regression modeling, “gbm” package’ were used to constructed the models. Briefly, 101 combinations of 10 machine learning algorithms were used to build the models based on a leave-one-out cross-validation (LOOCV) framework. Models with <3 genes were excluded. Simultaneously, each patient’s linear score and concordance index (C-index) are calculated based on various models, and the optimal model is selected based on the average highest C-index in the training and testing cohorts. Utilizing the optimal model, the DS for each patient was determined. See the supplementary methods table for details. The patients were then separated into high- and low-DS groups using the median DS value. Subsequently, Kaplan–Meier survival analysis was utilized to compare the OS rates of patients in various DS groups.

### Somatic mutation and CNV analysis

The “maftools” package was utilized to evaluate and visualize the mutation type and frequency of the genes (23). Correspondingly, the tumor mutation burden (TMB) of each LUAD sample was calculated based on the total count of somatic mutations per megabase (MB) in the exon coding region of the human genome. Different mutation types were classified as either synonymous or nonsynonymous mutations. The nonsynonymous variants included Frame_Shift_Del, Frame_Shift_Ins, In_Frame_Del, In_Frame_Ins, Missense, Nonsense, Nonstop, Splice_Site, and Translation_Start_Site. The maftools analysis focused on identifying significantly mutated genes (P <0.05) between the two groups and assessing the interaction effect of gene mutations. Only genes with mutations occurring 30 times or more in at least one group were considered for both analyses. GISTIC 2.0 was used to identify significant regions within CNV data (24). To quantify and compare CNVs, we calculated the fraction of altered genome (FGA), fraction of genome gained (FGG), and fraction of genome lost (FGL) for each sample. FGA reveals the proportion of genomic segments that have been altered. FGG/FGL considers specifically the genomic segments that have undergone gain or loss, respectively.

### Tumor microenvironment landscape and hallmark pathway analyses

TME at LUAD were evaluated under four aspects. First, the immune score, stromal score, ESTIMATE score, and tumor purity were calculated using the ESTIMATE algorithm (25). Secondly, three algorithms, Single Sample Gene Set Enrichment Analysis (ssGSEA), Tumor Immune Estimation Resource (TIMER, https://cistrome.shinyapps.io/timer/), and “MCPcounter” were used to quantify the relative infiltration of immune cells in the entire cohort (26–28). Thirdly, the seven steps of cancer immunity cycle were analyzed using the Tracking Tumor Immunophenotype (TIP) website (http://biocc.hrbmu.edu.cn/TIP/) (29). In the fourth step, 35 inhibitory immune checkpoints were extracted from a prior study. Subsequently, Gene Set Enrichment Analysis (GSEA) was utilized to identify underlying mechanisms in hallmark gene sets with the recommended criteria (FDR <0.25 and NES >1), in order to determine the underlying hallmark pathways associated with DS (30). Meanwhile, “Gene Set Variation Analysis” (GSVA) package was applied to the two DS groups with an adjusted p value <0.01 (31). The “h.all.v7.4.symbols.gmt” hallmark gene sets from the MSigDB database were employed for GSVA implementation.

### Assessment of immunotherapy and chemotherapy

To explore the predictive value of DS in LUAD patients after immunotherapy, we compared the immune dysfunction and exclusion (TIDE, http://tide.dfci.harvard.edu/) score; additionally, the submap algorithm was applied to compare the efficacy of immunotherapy among various DS subtypes (32, 33). In addition, four immunotherapy-treated cohorts, IMvigor210, GSE35640, GSE79671, and GSE173839, were collected to investigate the immunotherapy response ability of DS. The sensitivity of tumor cell lines to potential drugs was obtained from the Cancer Therapeutics Response Portal (CTRP, https://portals.broadinstitute.org/ctrp) and Profiling Relative Inhibition Simultaneously in Mixtures (PRISM, https://depmap.org/portal/prism/). The more sensitive a cell line is to a potential drug, the lower its area under the curve (AUC).

### Single-cell RNA-sequencing analysis

ScRNA-seq data was downloaded from GSE131907. Subsequently, the “Seurat” R package was utilized to measure the gene expression levels by processing the raw data from each sample (34). Cells with fewer than 200 detected genes were removed. Accordingly, the top 2000 highly variable genes were selected for subsequent clustering analysis. Following this, single cells were classified into distinct subgroups via the application of the FindNeighbors and FindClusters functions (dim = 15 and resolution =0.2). In addition, the tSNE was constructed utilizing the top 15 primary components. Subsequently, immune cells and tumor cells were identified using the “single R” and “copyKAT” packages (35, 36). Pseudotime trajectory analysis is a method used to study the temporal order of cells during development, differentiation, or other biological processes. Its goal is to reduce the dimensionality of single-cell data from high-dimensional to one-dimensional, thus representing the temporal changes of cells in a pseudotime manner. This aids in understanding the timeline of processes like differentiation, development, and transformation in tumor cells. Hence, the cell trajectory cancer cell populations were ordered in pseudotime using the “Monocle” package (37).

### Cell culture and transfection

Two human LUAD cells (PC-9 and H838) as well as a normal bronchial epithelial cell (BEAS-2B) were obtained from the American Type Culture Collection (Manassas, VA, USA). The PC-9 and H838 cells were grown in RPMI 1640 supplemented with 10% FBS and 1% penicillin-streptomycin, while BEAS-2B cells were grown in DMEM supplemented with 10% FBS and 1% penicillin-streptomycin in humidified air at 37°C and 5% CO_2_ (This condition simulates the physiological environment in the human body, aiding in maintaining normal cellular growth, metabolism, and function). To construct KYNU knockdown and negative control (NC) cell lines, H838 and PC-9 cells were seeded in 6-well plates at a density of 5 × 10^4^ cells/well and transfected with 50 nM siRNAs-KYNU and siRNA negative control (siRNA-NC) using lipofectamine 3000 (This reagent possesses high efficiency, broad spectrum, and low toxicity) following the manufacturer’s guidelines (Hanheng, Shanghai, China). After 48 h of transfection, subsequent experiments were conducted (Typically, after 48 hours of transfecting siRNA, the target gene’s expression is effectively disrupted in the vast majority of cases, while cells continue to maintain a relatively healthy and appropriate growth state). The siRNA sequences are provided in **Supplementary Table 3**.

### Tissue microarray and immunohistochemistry

For this study, a total of 15 TM tissue samples (HLugA030PG04-1) were utilized, including 15 LUAD and 15 adjacent non-tumor tissues. All the tissues were procured from Shanghai Outdo Biotech Co., Ltd. (Shanghai, China). TM was stained using IHC with KYNU. In brief, the antibodies were diluted to the suitable concentration and incubated overnight with the sections at 4°C. The avidin–biotin and streptavidin complex were then incubated with the biotinylated goat anti-rabbit IgG secondary antibody for 0.5 h. The cell nuclei were counterstained blue with Hematoxylin. Each specimen was graded based on the intensity (0: absent, 1: mild, 2: moderate, and 3: pronounced) and the proportion of positively stained cells (0: 0%, 1: 1%–25%, 2: 26%–50%, 3: 51%–75%, and 4: 76%–100%). The final IHC scores were then calculated as the product of the intensity score and the percentage score (38).

### RNA-isolation and real-time reverse transcription polymerase chain reaction

RNA extraction was performed employing RNA extraction kit (AG, Changsha, China) in accordance with the manufacturer’s instructions. The complementary DNA (cDNA) was synthesized from total RNA for each sample by using a reverse transcription kit (Promega, Madison, Wisconsin). Subsequently, the mRNA expression was quantitatively analyzed via RT-PCR (Roche Light Cycler^®^ 480II System). The relative gene expression was normalized to β-actin, acting as the control gene. The primer sequences for the genes are outlined in **Supplementary Table 4**. The fold change of the target gene was computed utilizing the 2^−ΔΔCt^ method.

### Proliferation assay

Cells in logarithmic phase were seeded into 96-well plates at density of 2,000 cells per 100 µl. They were then cultured for 0, 24, 48, 72, and 96 h. At the end of the incubation cycle, CCK-8 reagent (Saiku, Shanghai, China) was added to each well for 2 h at 37°C. Subsequently, the absorbance values of each well were measured at 450 nm. For colony formation assay, 500 cells were seeded into each well of a 12-well plate and incubated. After 10 days, the cells were stained with crystal violet solution, and fixed with 4% paraformaldehyde for 0.5 h. Finally, colonies consisting of more than 50 cells were counted.

### Migration and invasion assay

The transwell experiment was executed using a transwell chamber (Transwell, Corning Costar, USA). For migration assays, a total of 4 × 10^4^ cells were resuspended in 200 μl serum-free medium and placed to the upper chamber. The lower chamber was filled with 700 μl of medium containing 10% FBS. After 18 h, cells that migrated through the membrane were fixed and stained with hematoxylin. Following a 24 h-incubation period, cells that traversed the membrane were fixed with 4% paraformaldehyde for 30 minutes and stained with a crystal violet solution for 15 min. Subsequently, they were observed with a microscope, and five random fields of view were selected to count the cells. The protocol for the invasion assay was similar to the migration assay, with the exception that the upper chambers were coated with 70 μl of diluted Matrigel. In addition, the wound healing assay was utilized to examine cell migration. Serum-starved LUAD cells (2 × 10^5^) were seeded in 6 well plates and then transfected with siRNA-NC or siRNA-KYNU. Upon reaching >95% cell confluence, a sterile 20 μl pipette tip was used to create a scratch in the monolayer of cells. After a PBS wash, the cells were incubated for 0 and 24 h in the corresponding basic culture medium. The monolayer cells were examined microscopically, and the gap distance was measured quantitatively to ascertain LUAD cell migration.

### Statistical analysis

All statistical analyses and representations were conducted using R (version 4.2.1) and GraphPad Prism (version 9.00). The Chi-squared test was used to compare the proportion of individuals within two groups. Additionally, continuous variables in two or more groups were compared using Wilcoxon rank-sum test or Kruskal–Walli’s test. For correlation analysis, the Pearson correlation test was employed. A p-value less than 0.05 was deemed statistically significant.

## Results

### Genetic and transcriptional alterations of DRs in LUAD

**Figure 1:**
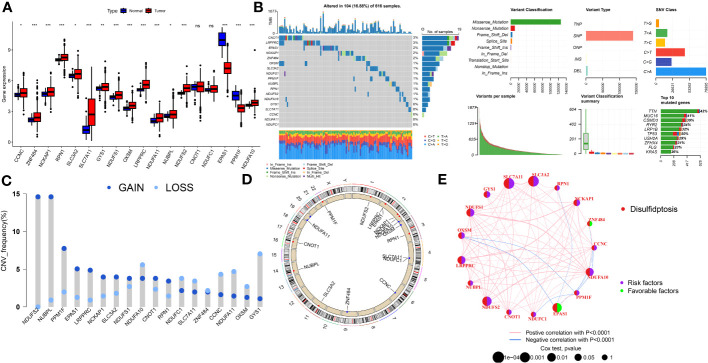
The genomic features and expression of DRs in LUAD. **(A)** The differential expression of DRs between tumor and normal samples. **(B)** Mutation landscape of DRs in TCGA-LUAD. **(C)** The CNV mutation frequency of DRs. **(D)** Chromosome position and alteration of DRs. **(E)** Molecular interaction network map of DRs Negative correlations are illustrated in green, while positive correlations are denoted in pink. ns, not significant, *P < 0.05, **P < 0.01, ***P < 0.001.

Gene expression analysis using bulk RNA-seq demonstrated that the majority of DRs displayed relative higher expression levels in LUAD tissues compared to para-carcinoma tissues (**Figure 1A**). As depicted in **Figure 1B**, 104 of 616 (16.88%) LUAD samples possessed genetic mutations. A total of 13 of 18 DRs were found to be mutated, with CNOT1 exhibiting the highest rate of mutation. Among them, missense mutations were found to be the most frequent (**Figure 1B**). In order to unmask the genetic modifications in DRs, we presented an overview of the frequency of somatic and copy number mutations with malignancies. Moreover, analysis of these 18 DRs revealed that CNV alterations were common. NDUFS2, NUBPL, PPM1F, EPAS1, and LRPPRC displayed widespread CNV amplification, whereas CCNC, NDUFA11, OXSM, and GYS1 showed widespread CNV deletions (**Figure 1C**). **Figure 1D** depicts the locations of CNV alterations in LUAD DRs. The correlation network composed of 18 DRs is illustrated in **Figure 1E** (correlation coefficient >0.4; the positive correlation is represented by the red line, negative correlation is represented by the blue line).

### Identification of DRs subtypes in the LUAD cohort

**Figure 2:**
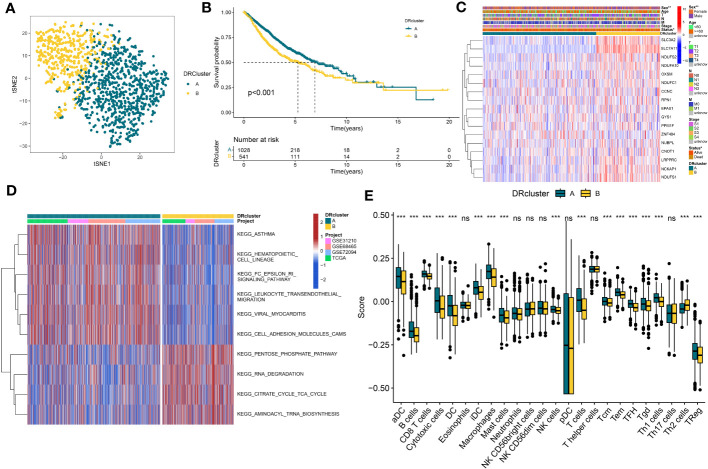
The construction of DRclusters. **(A)** tSNE plot of two DRclusters. **(B)** Kaplan-Meier survival analysis between two DRclusters. **(C)** Heatmaps of the distribution of DRs in the two DRclusters. **(D)** GSEA analysis indicating significant enrichment of pathways in the two DRclusters. **(E)** The proportion of 24 kinds of immune cells in two DRclusters. ns, not significant, *P < 0.05, **P < 0.01, ***P < 0.001.

In order to delve further into understanding the DRs’ expression pattern in the oncogenesis of LUAD, 1569 patients from 4 independent LUAD cohorts were included. Accordingly, we performed unsupervised clustering and classification on the combined LUAD cohort based on DRs. Our results showed that k = 2 was the optimal choice (**Figures S1**). In addition, the tSNE results revealed significant differences between the two clusters in terms of DRs expression (**Figure 2A**). Moreover, the Kaplan–Meier survival analysis demonstrated that the DRcluster A had a greater survival advantage than DRcluster B (**Figure 2B**). In addition, the clinicopathological characteristics of the various DR clusters also revealed significant differences (**Figure 2C**). To further explore the biological behavioral difference between these two clusters, we also conducted a GSVA enrichment analysis (**Figure 2D**). The results demonstrated that DRcluster A was primarily enriched for carcinogenic pathways like focal adhesion, EMC receptor interaction, and others. Moreover, **Figure 2E** data also revealed significant differences in the relative expression of immune infiltration cells across two DRclusters.

### Identification of genes subtypes and establishment of DS

**Figure 3:**
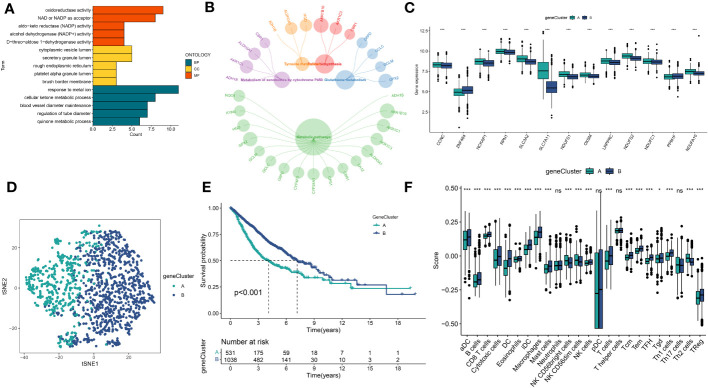
Identification of DRs gene clusters based on DEGs in DRclusters. **(A, B)** GO and KEGG enrichment analyses of DEGs among two DRclusters**(C)** Differences in the expression of DRs among the two genecluster **(D)** tSNE plot of two geneclusters. **(E)** Kaplan-Meier survival analysis between two geneclusters. **(F)** The proportion of 24 kinds of immune cells in two geneclusters. *p < 0.05; ***p < 0.001; ns, not significant.

**Figure 4:**
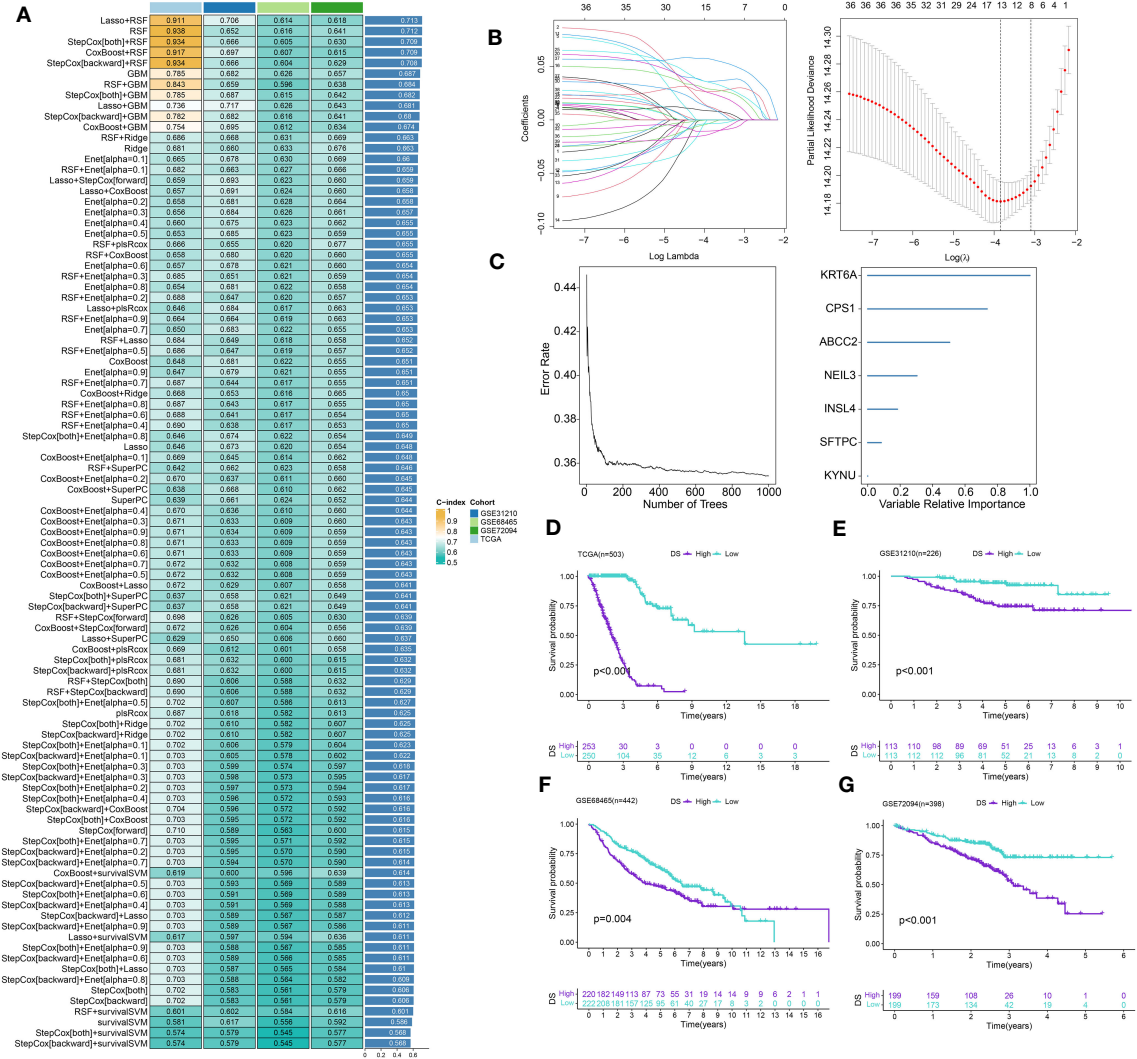
Construction of prognostic signature based on DEGs. **(A)** A total of 101 kinds of prediction models via a leave-one-out cross-validation framework and further calculated the C-index of each model. **(B)** Cvfit and lambda curves of LASSO regression applied with minimum criteria. **(C)** The number of trees determined by minimum error and importance of the four most valuable genes based on the RSF algorithm. **(D-G)** Kaplan-Meier survival curves of OS for high- and low-DS groups of patients in the TCGA, GSE31210, GSE68465, GSE72094 cohorts, respectively.

**Figure 5:**
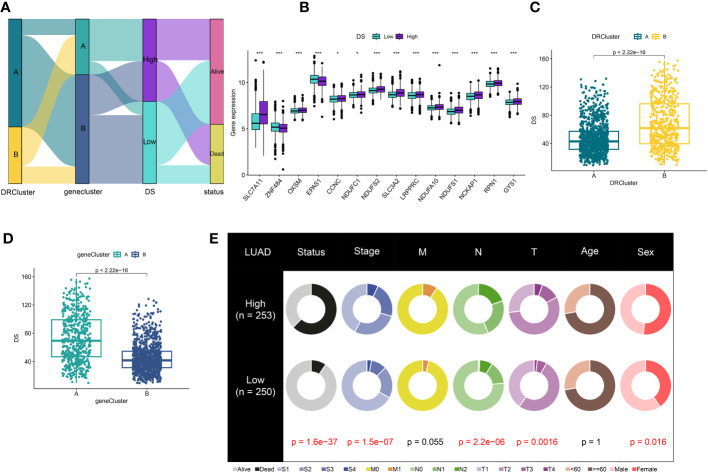
Correlation analysis of DS. **(A)** Alluvial diagram of clusters distributions in groups with different DS and survival outcomes **(B)** Expression of DRs between high- and low-DS groups. **(C)** Differences in DS between DRclusters. **(D)** Differences in DS between gene clusters. **(E)** The circular pie chart for the proportion difference of clinical indices. *P < 0.05, ***P < 0.001.

A total of 51 DEGs were identified from two DRclusters using the “limma” package. These DRs subtypes-related genes were significantly enriched in cellular metabolism (**Figures 3A, B**). Subsequently, we conducted a univariate Cox regression analysis for DEGs and identified 39 DEGs with prognostic importance for LUAD. To further investigate the heterogeneity of DRs subtypes, we performed unsupervised clustering on 39 DEGs. The 1569 patients with LUAD were also separated into two gene clusters, designated geneCluster A and B (**Figure S2**). Similar to DRcluster, these genes accurately differentiate LUAD patients, with distinct clusters of genes exhibiting variations in DRs, survival rates, and immune cell infiltration (**Figures 3C–F**). Following this, the LOOCV framework was used to fit 101 prediction models to both training and testing sets. The C-index for each model was calculated, and based on the model with the highest average C-index (0.713), “Lasso+RSF” was deemed the optimal model (**Figures 4A–C**, **Supplementary Table 5**). A DS was calculated for each patient based on the expression of 7 genes (KRT6A, NEIL3, KYNU, ABCC2, SFTPC, CPS1, and INSL4) weighted by their regression coefficients in the model, and patients were divided to high- or low‐DS groups based on the median cutoff point of DS (**Supplementary Table 6**). As evident from the K–M survival analysis, OS rates were significantly diminished in the high-DS group compared to the low-DS group (**Figures 4D–G**). Moreover, the relationship between different types of patients and their prognoses was analyzed (**Figure 5**), with results suggesting that a low DS was related to a better prognosis in all patient categories.

### Evaluation of the DS

**Figure 6:**
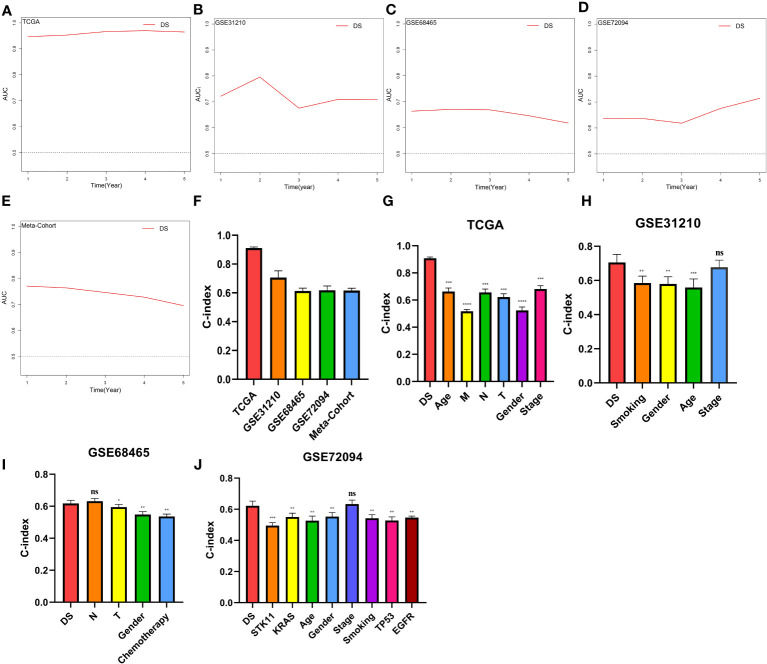
Evaluation of the DS. **(A-E)** Time-dependent ROC curves presented with the 1-5 year AUC in TCGA, GSE31210, GSE68465, GSE72094 and meta-cohort. **(F)** The C-index of the CDS for the TCGA, GSE31210, GSE68465, GSE72094 cohorts. **(G-J)** The C-index of the DS and other clinical factors in the TCGA, GSE31210, GSE68465, GSE72094 cohorts. *p < 0.05; **p < 0.01; ***p < 0.001; ****p < 0.0001; ns, not significant.

**Figure 7:**
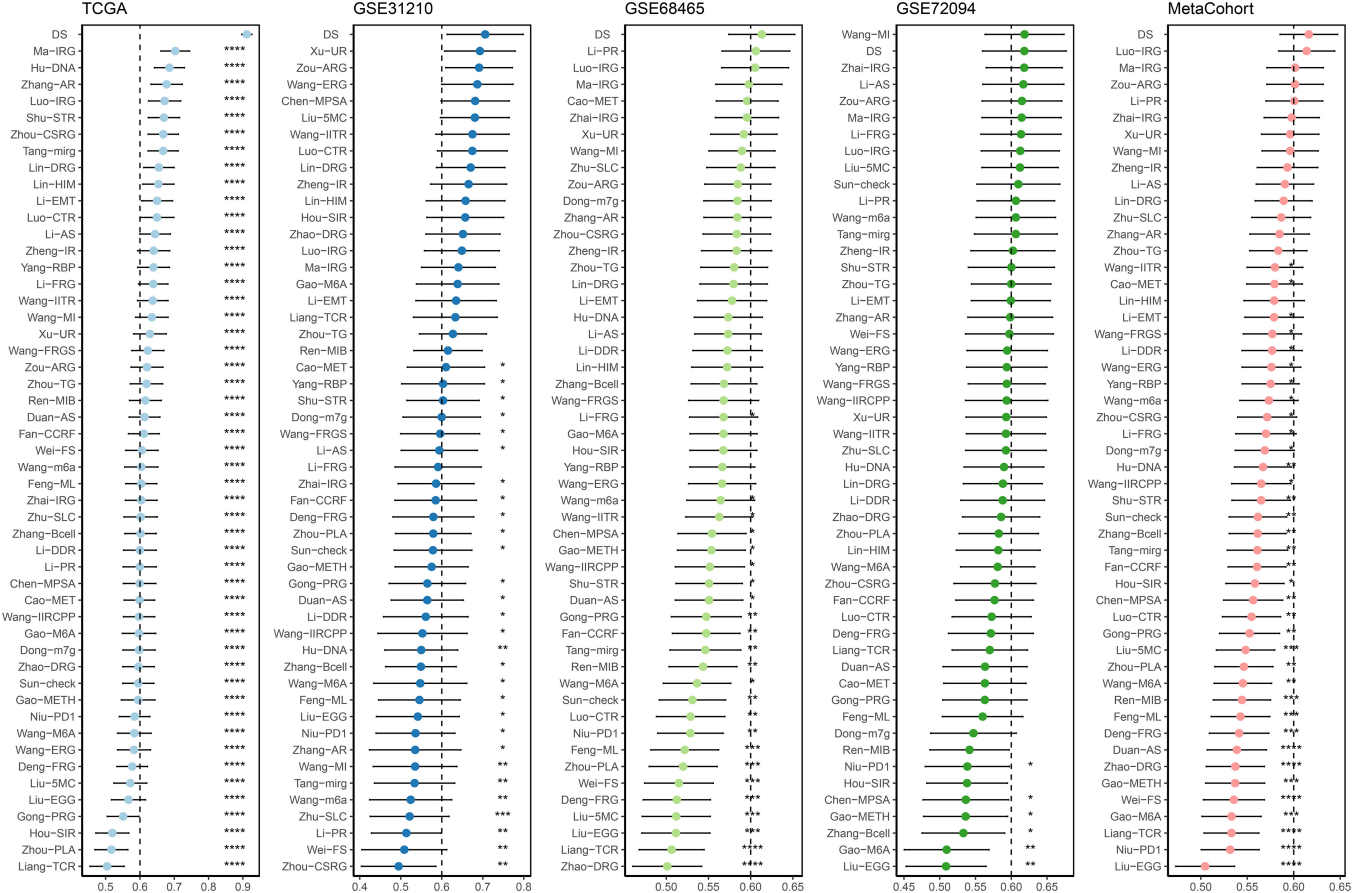
C-index analysis between the DS and 56 published signatures in TCGA, GSE31210, GSE68465, GSE72094 and meta-cohort. *p < 0.05; **p < 0.01; ***p < 0.001; ****p < 0.0001; ns, not significant.

A time-dependent receiver operating characteristic (ROC) curve was employed to assess the validity of DS, as the AUC value for TCGA (0.93–0.96), GSE31210 (0.68–0.79), GSE68465 (0.63–0.66), GSE72094 (0.63–0.72), and meta-cohort (0.71–0.78) (**Figures 6A–E**). In addition, the C-index of clinical factors in patients with LUAD was determined (**Figures 6F–J**). Notably, the DS had a higher predictive efficacy than the vast majority of clinical indicators. Subsequently, both DS and clinical indicators were subjected to univariate and multivariate Cox analyses. In all cohorts, the DS was determined to be an independent indicator of OS prognosis (**Tables 1**–**4**). In order to determine the prognostic efficacy of DS, we combined 56 previously published LUAD prognostic models and conducted a comparative analysis of each model’s C-index. These models were developed using a variety of biologically relevant features, including autophagy, EMT, ferroptosis, hypoxia, necroptosis, glycolysis, and m6A methylation. Accordingly, DS was found to exhibit superior performance relative to the vast majority of models across all cohorts (**Figure 7**, **Supplementary Table 7**). Cumulatively, these results, therefore, demonstrate that the DS would be a valuable LUAD prognostic model.

**Table 1:** Univariate and multivariate Cox analysis of the clinicopathological features and FA score with OS for TCGA cohort.

	Univariate Cox		Multivariate Cox	
Characteristics	HR(95%CI)	*P* value	HR(95%CI)	*P* value
Stage	1.977(1.586-2.463)	**< 0.001**	1.239(0.863-1.779)	0.245
M	1.727(1.18-2.527)	**0.005**	0.969(0.611-1.537)	0.895
N	1.942(1.575-2.394)	**< 0.001**	1.23(0.923-1.64)	0.157
T	1.816(1.386-2.38)	**< 0.001**	1.267(0.895-1.793)	0.183
Age	1.038(0.822-1.31)	0.754	NA	NA
Sex	1.041(0.847-1.28)	0.7	NA	NA
DS	0.118(0.084-0.165)	**< 0.001**	0.136(0.092-0.202)	**< 0.001**

Significant value is given in bold.

**Table 2:** Univariate and multivariate Cox analysis of the clinicopathological features and FA score with OS for GSE68465 cohort.

	Univariate Cox		Multivariate Cox	
Characteristics	HR(95%CI)	*P* value	HR(95%CI)	*P* value
N	2.029(1.689-2.438)	**< 0.001**	1.92(1.578-2.335)	**< 0.001**
T	2.062(1.587-2.68)	**< 0.001**	1.851(1.403-2.442)	**< 0.001**
Gender	1.262(1.051-1.516)	**0.013**	1.236(1.018-1.5)	**0.032**
Chemotherapy	1.412(1.15-1.734)	**< 0.001**	1.279(1.032-1.586)	**0.024**
DS	0.767(0.639-0.92)	**0.004**	0.819(0.678-0.989)	**0.038**

Significant value is given in bold.

**Table 3:** Univariate and multivariate Cox analysis of the clinicopathological features and FA score with OS for GSE31210 cohort.

	Univariate Cox		Multivariate Cox	
Characteristics	HR(95%CI)	*P* value	HR(95%CI)	*P* value
Smoking	1.417(0.882-2.277)	0.15	NA	NA
Gender	1.344(0.839-2.152)	0.219	NA	NA
Age	1.263(0.777-2.052)	0.346	NA	NA
Stage	2.774(1.732-4.441)	**< 0.001**	2.305(1.417-3.75)	**< 0.001**
DS	0.4(0.229-0.7)	**0.001**	0.495(0.278-0.881)	**0.017**

Significant value is given in bold.

**Table 4:** Univariate and multivariate Cox analysis of the clinicopathological features and FA score with OS for GSE72094 cohort.

	Univariate Cox		Multivariate Cox	
Characteristics	HR(95%CI)	*P* value	HR(95%CI)	*P* value
STK11	1.028(0.72-1.469)	0.879	NA	NA
KRAS	0.767(0.588-0.999)	**0.049**	0.911(0.693-1.198)	0.506
Age	1.258(0.836-1.894)	0.27	NA	NA
Gender	0.733(0.564-0.952)	**0.02**	0.746(0.569-0.979)	**0.035**
Stage	1.969(1.477-2.625)	**< 0.001**	1.956(1.459-2.623)	**< 0.001**
Smoking	1.248(0.694-2.245)	0.459	NA	NA
TP53	0.861(0.645-1.151)	0.313	NA	NA
EGFR	2.58(1.274-5.226)	**0.008**	2.025(0.986-4.159)	0.055
DS	0.549(0.416-0.724)	**< 0.001**	0.619(0.465-0.825)	**0.001**

Significant value is given in bold.

### Comparison of the mutations and CNV between DS groups

**Figure 8:**
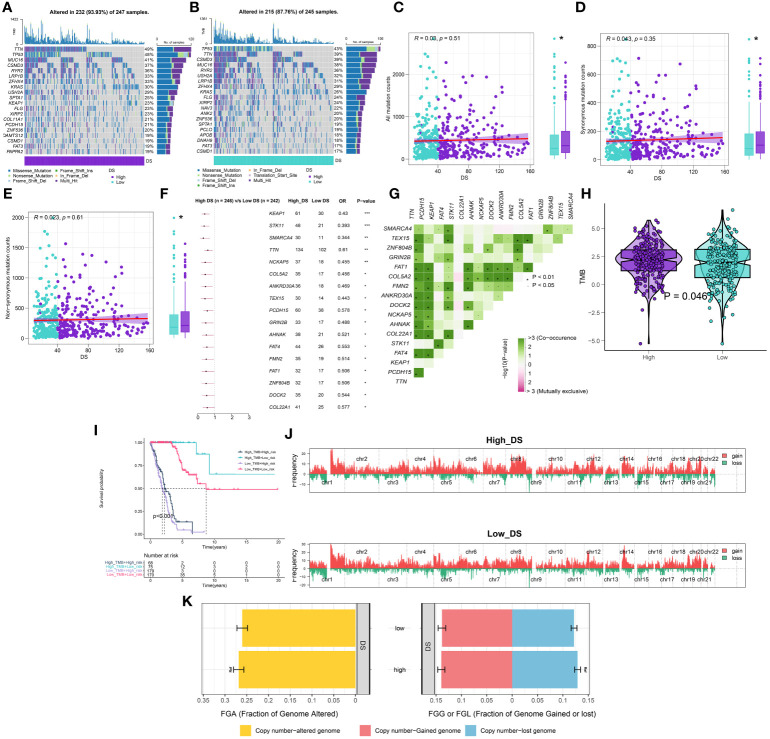
Integrated comparisons of somatic mutation and CNVs between high- and low-DS groups in the TCGA cohort. **(A, B)** Visual summary showing common genetic alterations in the high and low-DS groups. **(C-E)** Association between all mutation counts, synonymous mutation counts, nonsynonymous mutation counts, and DS and their distribution in the DS groups. **(F)** Forest plot of gene mutations in the patients. **(G)** Interaction effect of genes mutating differentially in patients. **(H)** Tumor mutation burden between high- and low-DS groups. **(I)** Comprehensive survival analysis based on DS and TMB. **(J)** Gene fragments profiles with amplification (red) and deletion (green) among the DS groups. **(K)** Comparison of the fraction of the genome altered, lost, and gained between the DS groups. *P < 0.05, **P < 0.01, ***P < 0.001.

Using the “maftool” package, a comparison was made between the distribution differences of somatic mutations observed in high- and low-DS groups. (**Figures 8A, B** **)**. Comparing the frequency of mutants between the high- and low-DS groups, more somatic mutations, both synonymous and nonsynonymous, were observed in the high-DS group (**Figures 8C–E**). In addition, maftools analysis results showed that 17 genes mutated more frequently in LUAD patients in the high-DS group, including KEAP1, STK11, SMARCA4, TTN, NCKAP5, COL5A2, ANKRD30A, TEX15, PCDH15, GRIN2B, AHNAK, FAT4, FMN2, FAT1, ZNF804B, DOCK2 and COL22A1 (**Figure 8F**), and there was extensive co-mutation between these genes (**Figure 8G**). This is consistent with the mutation analysis described above; accordingly, TMB was found to be higher in the high-DS group compared to the low-DS group (**Figure 8H**). Subsequently, LUAD patients were classified into two mutation groups based on their TMB score. When combining DS and TMB, we discovered that patients with low TMB from the high-DS group had the worst prognosis (**Figure 8I**). Subsequently, we used the GISTIC 2.0 software to decipher the amplification and deletion of CNA on chromosome. Compared to the high-DS group, the low-DS group had a greater burden of amplification and deletion at both the arm and focal levels (**Figure 8J**). However, no significant disparities were observed between the high- and low-DS groups in terms of FGA, FGG, and FGL (**Figure 8K**). These results indicate a certain degree of correlation between DS and mutations.

### TME analysis

**Figure 9:**
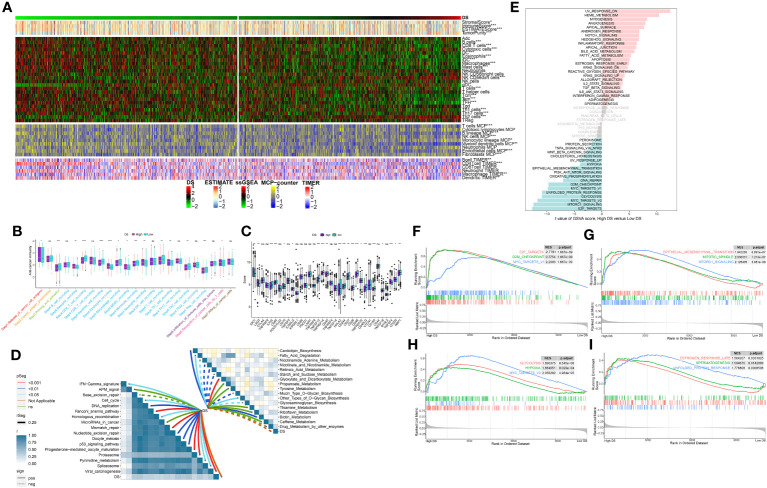
Analysis of the TME in different DS groups. **(A)** Differences in immune infiltration status between two DS groups were evaluated by four algorithms. **(B)** The differences of cancer immunity cycle were showed in boxplot between two DS groups. **(C)** The differences of immune checkpoint related genes were showed in boxplot between two DS groups. **(D)** The correlations between the TIIClnc signature score and metabolic immune-related pathways, immune-related pathways based on GSVA of GO and KEGG terms were displayed in butterfly plot. **(E)** The difference in the hallmark gene sets between different DS groups based on GSVA. **(F-I)** The GSEA results for the 12 overlapping upregulated hallmark pathways in terms of the high-DS group. *p < 0.05; **p < 0.01; ***p < 0.001; ****p < 0.0001; ns, not significant.

In order to evaluate the discriminative potential of the DS subgroup for the TME and its applicability in immunotherapy, we simultaneously evaluated the abundance of immune cell infiltration across multiple samples using four distinct algorithms. Unsurprisingly, as the DS increased, the number of immune cells declined (**Figure 9A**). Apparently, the activation of key steps in the cancer immunity cycle, such as step 3 (priming_and_activation) and step 4 (CD4 T cell recruiting, Dendritic cell recruiting, Macrophage recruiting, Monocyte recruiting, and T cell recruiting) appeared to be significantly higher in the low-DS group than in the high-DS group (**Figure 9B**). Subsequently, the expression profile of immune checkpoints in the two DS groups were further evaluated. Accordingly, the analysis revealed that the low-DS group demonstrated elevated expression levels of immune checkpoints, including HHLA2 and CD48 (**Figure 9C**). Given the observed upregulation of immune-related characteristics in the group with low DS, its underlying biological mechanisms were investigated further. As evident from the findings, DS exhibited correlations with multiple metabolic pathways (**Figure 9D**). In addition, DS demonstrated a strong correlation with numerous immunotherapeutic strategies (**Figure 9D**). To investigate the cancer signaling pathways associated with DS, GSVA analysis was performed on high- and low-DS groups. Using a predetermined threshold, we discovered that 16 signature pathways were significantly upregulated in the high-DS group compared to the low-DS group (**Figure 9E**). GSEA validated that 12 of these pathways were upregulated in high-DS patients, the majority of which are known to be carcinogenic (**Figures 9F–I**).

### Assessment of immunotherapy and chemotherapy

**Figure 10:**
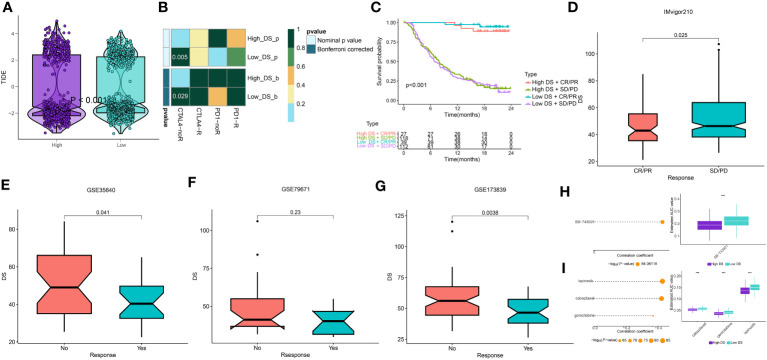
Prediction of immunotherapy and chemotherapy response. **(A)** A violin diagram illustrates the variance in TIDE scores among patients with diverse DS. **(B)** A comprehensive submap analysis of the meta-cohort and melanoma patients, inclusive of intricate immunotherapeutic data. **(C)** A Kaplan-Meier plot delineates the survival rates for patients categorized into high- and low-DS groups within the IMvigor cohort. **(D-G)** A box diagram depicts the disparity in DS among patients exhibiting immunotherapy responses in the IMvigor210, GSE35640, GSE79671, and GSE173839 cohorts. **(H, I)** The findings from the correlation study and differential drug response analysis of CTRP-derived pharmaceuticals and PRISM-derived pharmaceuticals are presented. ***P < 0.001.

The potential of an immunotherapy response was subsequently predicted for each immune cluster utilizing the TIDE algorithm and submap analysis. Accordingly, lower TIDE scores were observed in the low-DS group, implying a higher sensitivity to immunotherapy in these patients (**Figure 10A**). Moreover, the submap results indicated that the group with a low DS level was more sensitive to CTLA4 inhibitors (**Figure 10B**). Although we evaluated an individual’s immunotherapy efficacy using two algorithms, it remains critical to directly compare the curative efficacy of immunotherapy cohorts across various DS groups. As a result, four immunotherapy cohorts were included for further analysis. In the IMvigor210 cohort, patients with DR/PR had significantly longer OS compared to patients with SD/PD, whereas the influence of DS on patient prognosis was minimal (**Figure 10C**). However, patients who responded better to immunotherapy had lower DS levels across all cohorts (**Figures 10D–G**). Although there were no significant differences in patient survival and DS between the two groups for some cohorts, the propensity for these results was consistent for the other cohorts. In addition, these results demonstrate that the DS was able to predict the efficacy of ICBs and can provide direction for the deployment of immunotherapy. Thus, to pinpoint candidate drugs that may exhibit heightened sensitivity in LUAD patients, we conducted drug response predictions using CTRP- and PRISM-derived data. Finally, the cross-correlation of the two pharmacogenomics databases allowed us to predict four drugs or compounds (including SB−743921, ispinesib, cabazitaxel, and gemcitabine) with therapeutic potential in patients (**Figures 10H, I**).

### Single-cell sequencing analysis

**Figure 11:**
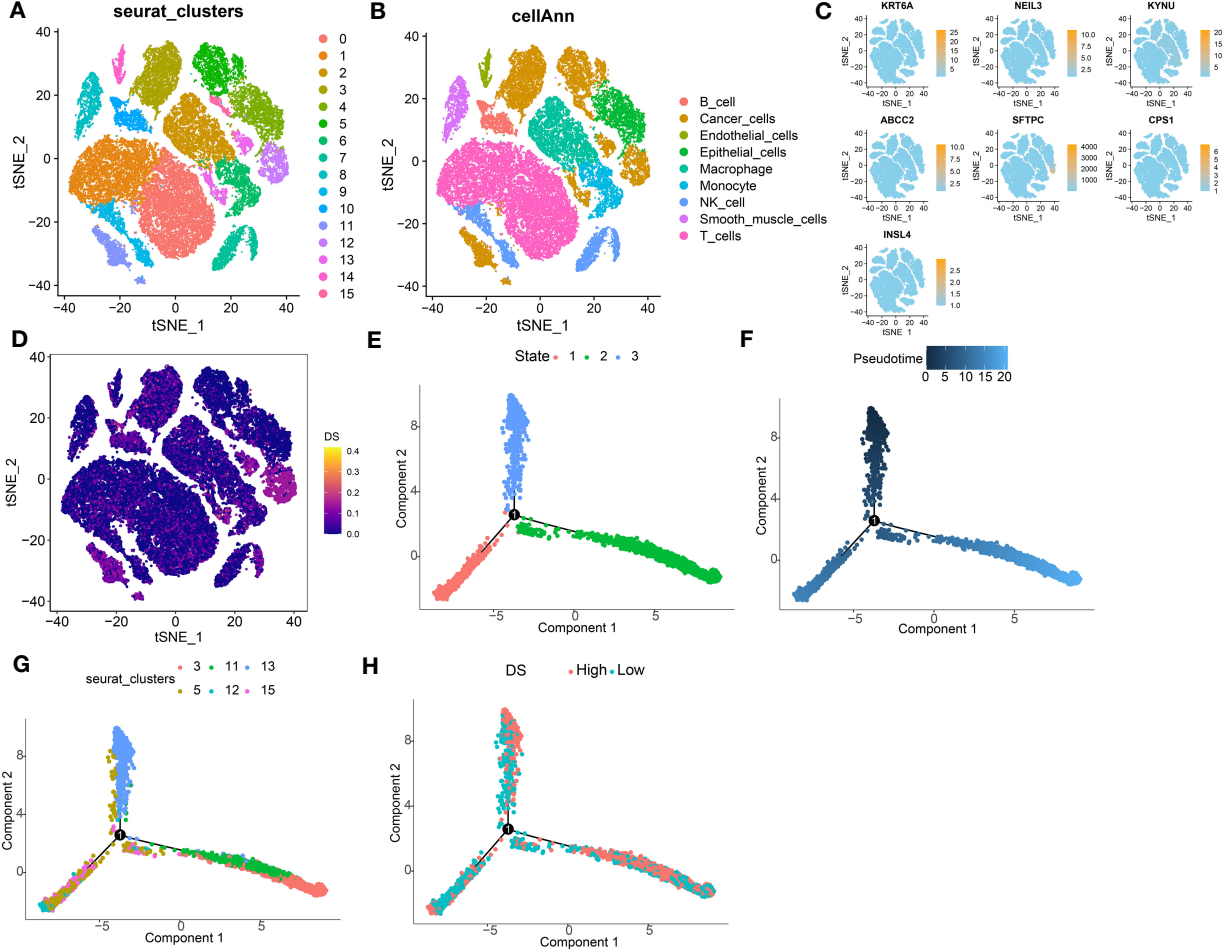
Exploration of DS in LUAD scRNA-seq data. **(A)** t-SNE plot colored by 16 cell subpopulations. **(B)** t-SNE plot of the distribution of 9 cell types. **(C, D)** Evaluation of DS gene expression and DS in scRNA-seq data in scRNA-seq data. **(E-H)** Pseudotime trajectory analysis in LUAD cells (Cells are colored based on states, pseudotime, cluster and DS groups.

To analyze the expression of DS in TME, we used the LUAD single-cell dataset GSE131907 from the GEO database. All the cells were partitioned into 16 clusters using the k-nearest neighbor (KNN) clustering algorithm (**Figure 11A**). Subsequently, using the “single R” and “copycat” packages to annotate all cells, we were able to identify 9 distinct cellular subtypes, including B cells, endothelial cells, epithelial cells, cancer cells, macrophage cells, monocyte cells, smooth muscle cells, NK cells, and T cells (**Figure 11B**). Most of these cells are important components of the TME mentioned in the above results. Subsequently, we investigated the single-cell transcriptome localization of 7 genes in DS (**Figure 11C**). Concurrently, the DS for each cell was calculated, which showed that cells with a high DS predominately resided in the region of cancer cells (**Figure 11D**). Additionally, the temporal sequence of cancer cellular differentiation was revealed by the analysis of the pseudotime trajectory. Accordingly, low DS cancer cells appear at an earlier pseudotime than high DS cancer cells, which are primarily found in the earliest stages of differentiation (**Figures 11E–H**).

### KYNU evaluation in the LUAD cells

**Figure 12:**
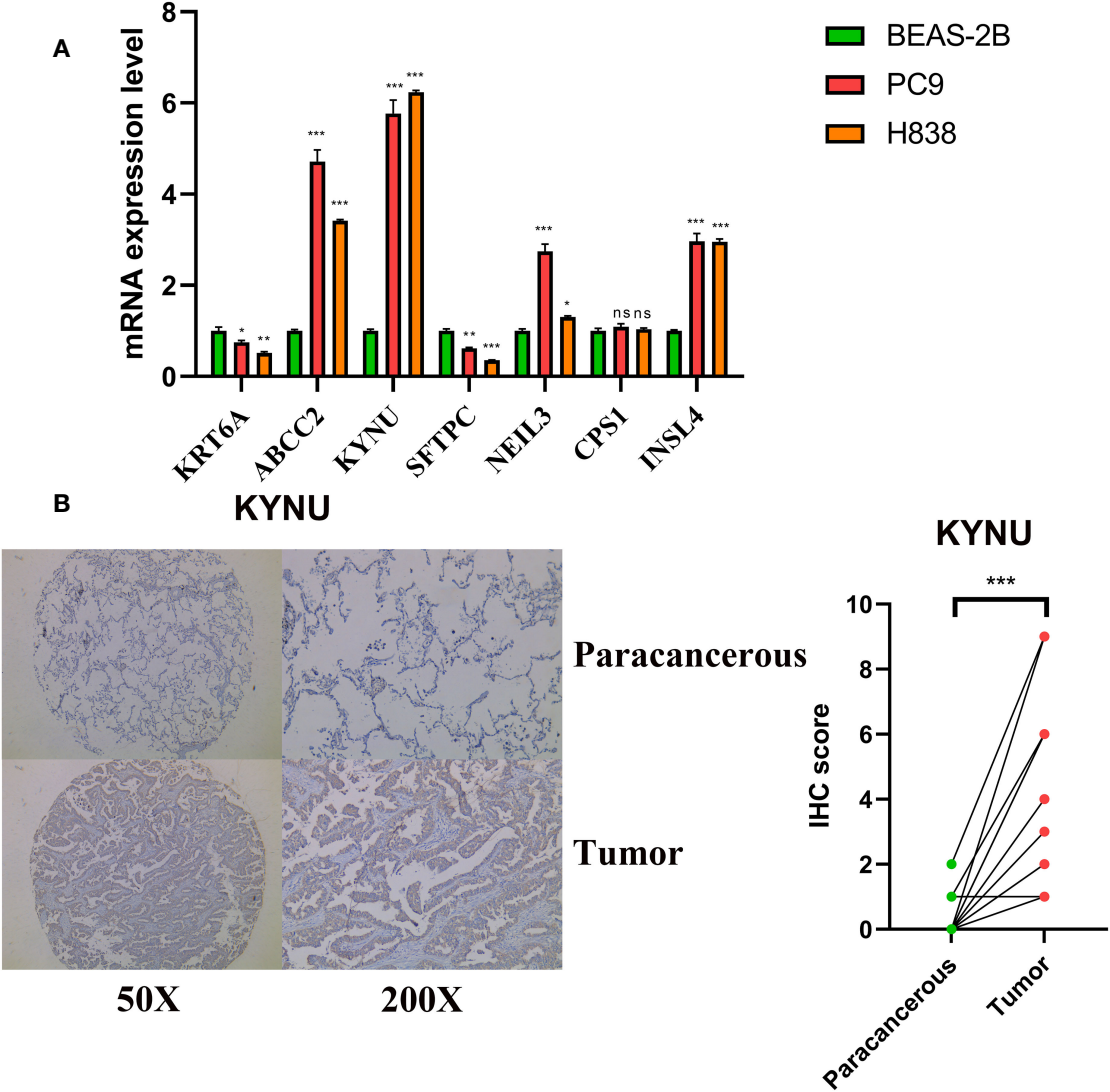
Validation of expression levels of DS genes. **(A)** DS genes expression in LUAD and normal cell lines. **(B)** Protein expression levels of KYNU were assessed by IHC. *p < 0.05; **p < 0.01; ***p < 0.001; ns, not significant.

**Figure 13:**
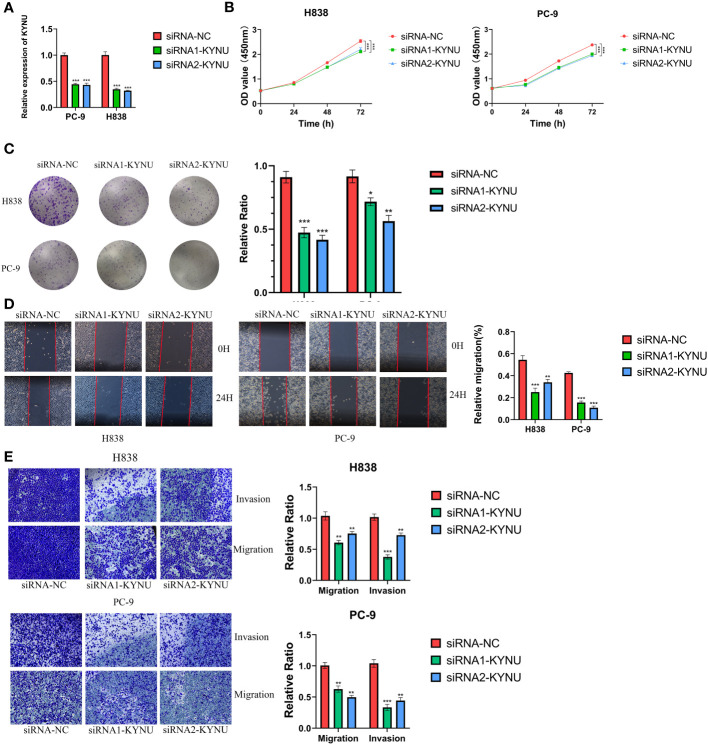
**(A)** The effect of siRNA to knockdown KYNU in LUAD cell lines was measured by RT–qPCR. **(B, C)** The CCK-8 and clone formation assays showed that knockdown of KYNU inhibited the proliferation of LUAD cells. **(D, E)** The wound healing and transwell assays showed that knockdown of KYNU inhibited the migration and invasion of LUAD cells. *P < 0.05; **P < 0.01; ***P < 0.001.

The qRT-PCR experiments were performed on LUAD cell lines to confirm the expression levels of DS genes in LUAD. Our finding discovered that ABCC2, NEIL3, KYNU, and CPS1 exhibited elevated expression in LUAD cell lines, while KRT6A and SFTPC were found to be underexpressed (**Figure 12A**). In addition, KYNU exhibited the most significant correlation between high expression and unfavorable poor patient prognosis. No reports have documented the role of KYNU in LUAD to date. As a result of these considerations, KYNU was selected as the focus of further experiments. Correspondingly, IHC staining analysis showed that the protein level of KYNU expression was elevated in LUAD tumor tissues relative to paracancerous tissues (**Figure 12B**). Subsequently, we investigated the function of KYNU in PC-9 and H838 LUAD cell lines through a series of cell-based experiments. Initially, the effect of the siRNA was confirmed via RT-qPCR (**Figure 13A**). As demonstrated by CCK-8 and clone formation assays, KYNU knockdown inhibits LUAD cell growth and their clone formation capacity (**Figures 13B, C**). In addition, wound healing and transwell assays confirmed that KYNU knockdown inhibited the cell migration and invasion capabilities of LUAD cells (**Figures 13D, E**). The precision of these findings corroborated that the expression of KYNU mirrored the variations anticipated through bioinformatic prediction.

## Discussion

LUAD persistently remains the principal contributor to cancer-related deaths among all cancer types and poses a substantial threat to global health (39). Prior research has revealed that the onset and progression of LUAD involve complex biological mechanisms, such as a multitude of genetic and epigenetic modifications (40, 41). Numerous staging systems have been proposed and utilized for clinical determinations to predict patient prognoses; however, these systems predominantly rely on clinicopathological features, ignoring the critical influence of complex molecular pathogenic processes in the oncogenesis and progression of LUAD (42, 43). As a result, there have been negligible improvements in patient outcomes. Consequently, the identification of superior predictive biomarkers for treatment responsiveness and patient outcomes could be advantageous in optimizing individualized therapeutic strategies and prognostic management for those afflicted with LUAD. Atypical accumulation of intracellular disulfides induces disulfide stress, which results in cellular toxicity and ultimately induces cell death (16, 44). Disulfide bonds are the most important redox-reactive covalent bonds between two cysteine residues within proteins. These bonds are regarded as cellular redox regulators and are intimately linked to the formation of disulfides. Recent studies have revealed that neoplastic cells may also experience disturbances in disulfide metabolism as a result of oxidative stress, a process that can potentially inhibit the proliferation of tumor cells and induce their apoptosis (45, 46). In addition, the disulfides inherent to neoplastic cells may act as conduits, modulating the responsiveness to chemotherapeutic agents and immunotherapy, and possibly serving as prognostic markers (47–79). This suggests that the application of disulfidptosis-focused translational medicine holds considerable promise as a candidate for clinical implementation across an array of human malignancies. Several DRs have been implicated in pathological and physiological processes of a variety of tumor. To counterbalance the oxidative stress induced by their heightened metabolic rate, tumor cells can upregulate the expression of the catalytic subunit SLC7A11 of the Xc−system, thereby maintaining high levels of glutathione (50). In addition, the overexpression of SLC7A11 in glioma cells improves their resistance to oxidative stress and decreases their sensitivity to temozolomide (51). In this regard, SLC3A2 is significantly upregulated in several types of malignant tumor cells, including those of the lung, breast, and prostate (52, 53). Furthermore, SLC3A2 is also an independent prognostic indicator for thymic epithelial tumors and NSCLC (54). Consequently, the overexpression of SLC3A2 contributes to radiotherapy resistance in tumors, indicating that SLC3A2 could surface as a promising clinical prospect in cancer treatment (55). Moreover, models composed of DRs have been established in certain tumor types, demonstrating their potential predictive value for patient prognosis and treatment efficacy (56, 57). These findings indicate that DRs have significant potential for elucidating the molecular mechanisms underlying LUAD and identifying novel biomarkers. Nonetheless, there is a dearth of pertinent research on how DRs influence prognosis, immune infiltration, and clinical response in LUAD.

In this study, we first analyzed the characteristics of DRs in LUAD, including extensive genetic and transcriptional level alterations. The majority of these genes are upregulated in LUAD patients and are associated with a poorer prognosis, suggesting a plausible role for DRs in the pathogenesis of LUAD. Using unsupervised clustering techniques on DRs transcriptomic expression data, we then divided LUAD patients from four distinct cohorts into two subgroups, designated DRcluster A and DRcluster B. In DRcluster B, the majority of DRs were significantly upregulated, indicating relatively active disulfidptosis. Compared to patients in DRcluster A, DRcluster B was associated with an increase in the number of immune cells that infiltrated the affected tissue. Significant infiltration levels of effector immune cells are critical for a successful immunotherapeutic response. Typically, a higher CD8+ T cell infiltration rate is indicative of a better prognosis for survival. As evidence, a higher concentration of cytotoxic CD8+ T cells permeating the tumor has been linked to superior outcomes for patients with NSCLC (58). This is consistent with our prognosis and analysis results of immune infiltration. As a result, we proceeded to identify 86 DEGs that distinguished the two DRclusters, and based on these DEGs, we formed a pair of gene clusters. Intriguingly, we discovered statistically significant differences in OS, DRs, and TME between gene clusters, revealing a strong correlation between DRclusters and gene clusters. In light of the lack of DRclusters for clinical application and the paucity of biomarkers for prognosis tracking, we developed a robust and effective model by transforming 10 machine learning algorithms into more than 101 combinations and selecting the best performing algorithm determined by the mean C-index across four LUAD cohorts (59). This facilitated the creation of a robust and efficient DR-based prognostic model, suitable for appraising the prognosis of LUAD patients. Ultimately, the combination of LASSO and RSF was deemed the superior model for constructing the DS. Survival analysis utilizing the median value of the DS revealed its association with LUAD prognosis, and concordant results were obtained from three independent cohorts. The AUC at various time points and the C-index suggest that the DS has exceptional clinical efficacy, surpassing the performance of a substantial number of other clinical attributes. Significantly, when compared to the 56 previously reported molecular signatures for LUAD, the predictive performance of the DS is consistently superior in nearly all cohorts examined.

The composition of our DS comprises 7 genes, including KRT6A, NEIL3, KYNU, ABCC2, SFTPC, CPS1, and INSL4. In addition, qRT-PCR analysis revealed that LUAD and human bronchial epithelial cell expression of the majority of genes differed significantly. Numerous identified genes exhibit a strong correlation with the onset and advancement of LC. For instance, overexpression of KRT6A in NSCLC is associated with poor prognosis (60). KRT6A, acting downstream of LSD1, upregulates G6PD and the pentose phosphate pathway flux via the MYC signaling cascade, thereby promoting NSCLC growth and invasion (61). The upregulation of NEIL3 expression in NSCLC tissues and cell lines correlates with clinical progression and a poor prognosis. By partially activating the PI3K/AKT/mTOR signaling pathway, NEIL3 contributes to the progression of NSCLC (62). A recent study demonstrated that SFTPC expression is suppressed in human LUAD tissues and cell lines, and its overexpression inhibits LCcell proliferation *in vitro* and *in vivo* (63). INSL4, via autocrine or paracrine effects, promotes the proliferation and invasion of NSCLC by enhancing the MAPK and AKT signaling pathways. Moreover, INSL4 serves as a detrimental prognostic indicator for patients suffering from NSCLC. Among the 7 genes, IHC confirmed the elevated expression of KYNU in LUAD tissue samples, and the association between high KYNU expressions was notably associated with a shorter OS in the cohort. Moreover, cellular experiments suggest that the knockdown of KYNU can curb the proliferation, invasion, and migration capabilities of LUAD cells, suggesting its oncogenic role in LUAD.

TMB has recently emerged as a promising prognostic biomarker for numerous tumor types. A higher TMB is frequently associated with improved survival outcomes (9). For instance, a study of NSCLC patients revealed that those with elevated TMB levels experienced prolonged OS when subjected to PD-1/PD-L1 antibody therapy (64). In this study, LUAD patients with an escalated DS had elevated TMB. This could be the result of patient heterogeneity or a small sample size. Recently, the proliferation and efficacy of targeted immunotherapies have begun to transform the landscape of cancer treatment (65, 66). Given the complex interaction between the tumor immune microenvironment and host immune responses, there is an urgent need for predictive biomarkers that facilitate individualized therapy. Increased concentrations of CD8+ T cells in the tumor microenvironment correlate with an improved prognosis and increased survival rates among patients with NSCLC (67). Furthermore, the existence of dysfunctional CD8+ T cells within lung tumors and malignant pleural effusions has been documented, thereby diminishing their capacity to mount an effective antitumor response (68). In preclinical studies, NK cell-based therapies have demonstrated the ability to prevent the development of pulmonary metastases (69). Several studies demonstrate that extracorporeal stimulation of autologous Natural Killer (NK) cells with Interleukin-2 (IL-2) in conjunction with adoptive transfer and subcutaneous IL-2 infusions increased overall survival (OS) in a subset of patients with advanced cancers (70). Within the pulmonary environment, neoplasm-associated B lymphocytes can differentiate into plasma cells, thereby producing tumor-specific antibodies capable of recognizing and reacting to tumor-associated antigens (71). Accordingly, the presence of both follicular B cells and tumor-infiltrating plasma cells has been positively correlated with increased longevity in patients with NSCLC, highlighting the protective contribution of plasma cells and antibodies in combating tumor proliferation (72). Moreover, single-cell sequencing analysis results indicate that high DS cells are primarily located in the tumor cell and B cell regions, suggesting a possible interaction between them. Consistent with previous findings, LUAD patients with a low DS demonstrated a high level of immune cell infiltration, including CD8 cells, NK cells, B cells, dendritic cells, mast cells, and central memory T cells, all of which play crucial roles in either bolster or counter tumor immunity during immunotherapy. Tumor cells characterized by reduced differentiation levels frequently demonstrate accelerated growth rates and heightened invasiveness, often correlating with unfavorable prognoses (73). Pseudotime analysis outcomes revealed a spatial disposition wherein tumor cells exhibiting diminished CDS levels occupied the initial phase of the differentiation trajectory. Conversely, those with elevated CDS levels were situated at the concluding stage of differentiation. Consequently, it becomes evident that CDS levels could potentially be linked to the extent of differentiation as well as invasiveness in tumor cells. In accordance with the tumor immunoediting hypothesis, the high-DS group exhibited greater immunosuppression but decreased immunoreactivity compared to the low-DS group (74). Disparities in the immune infiltrating microenvironment could potentially contribute to cancer progression and result in a poorer prognosis. Elevated IDO1 expression contributes to the development of an immunosuppressive TME by promoting T cell and NK cell inactivation and activating and expanding Tregs and DCs (75, 76). The role of IDO1-mediated tryptophan (TRP) metabolism in resistance to therapies targeting CTLA-4 or PD-1 demonstrates its potential as a promising target to augment existing immunotherapy approaches (77). CD40 is an essential co-stimulatory protein involved in the pro-inflammatory immune activation of antigen-presenting cells like dendritic cells and immunosuppressive macrophages within the cancer landscape (78, 79). Earlier investigations demonstrate that CD40 stimulation, in addition to activating tumor-associated immunosuppressive macrophages and T cells and inhibiting tumor progression (80, 81), also remodels the TME and heightens the tumor’s responsiveness to checkpoint blockade therapies in various types of cancer (82, 83). The findings of the current study show that the expression of IDO1 was downregulated in the low-DS group relative to the high-DS group, while CD40 expression was upregulated. In addition, differences in the expression of other immune checkpoints between the two groups suggested immunotherapy would have divergent effects (84). To determine the degree of immunotherapy response in LUAD patients, we evaluated the TIDE and submap algorithms to discovered that low-DS individuals identified by the model may be suitable candidates for immune checkpoint blockade therapies targeting CTLA-4 and PD-1. The scores based on immunotherapy algorithm scores are merely a reflection of theoretical hypotheses and cannot represent the actual efficacy within actual cohorts. For a more comprehensive analysis of the predictive efficacy of DS in immunotherapy, we thus incorporated multiple immunotherapy cohorts. Thus, in predicting therapeutic responses within immunotherapy cohorts, DS exhibits a trend that is consistent with immunotherapy algorithms, as indicated by our findings. These results suggest that a lower DS score may be a potent indicator of immunotherapy response in patients with LUAD.

The efficacy of pharmaceutical interventions is closely correlated with drug sensitivity and individual variation, indicating that personalized therapies based on specific subtypes could reduce the prevalence of ineffective therapies among LUAD patients. An analysis of drug sensitivity differences among LUAD patients with varying DS revealed 4 pharmaceuticals with significantly divergent sensitivities. Subsequent sensitivity projections demonstrated that SB-743921, cabazitaxel, gemcitabine, and ispinesib could potentially serve as superior therapeutic options for individuals with a high DS. Through signaling pathways such as G2M_CHECKPOINT, DNA_REPAIR, and PI3K_AKT_MTOR_SIGNALING, some of these pharmaceuticals exert their antitumor effects, as revealed by our enrichment analyses. Cabazitaxel functions by closely interacting with microtubule proteins, resulting in the inhibition of their depolymerization and consequently the inhibition of cellular mitosis. This mechanism induces cell cycle cessation, resulting in the programmed death or apoptosis of neoplastic cells (85). Recent research revealed that cabazitaxel can induce G2/M phase block and autophagy in LUAD cells by inhibiting the PI3K-AKT-mTOR pathway, indicating its potential as a chemotherapy drug for LUAD patients (86). Gemcitabine is a pyrimidine nucleoside analogue antimetabolite that can inhibit the synthesis and repair of DNA, thereby inducing cellular autophagy and apoptosis (87). When used as a stand-alone treatment, gemcitabine has consistently demonstrated response rates greater than 20% while maintaining a favorable tolerability profile. Moreover, its therapeutic efficacy can be augmented through combination regimens with platinum-based compounds such as cisplatin, thereby synergistically enhancing its overall efficacy (88, 89).

Despite the use of potent open-source data to elucidate two distinct characteristics of LUAD disulfidptosis subtypes and to develop a robust DS evaluation model, this investigation is limited by certain factors. First, this study relies on patient data obtained from publicly available retrospective cohorts and lacks the prospective real-world data required to validate the clinical applicability of the proposed scoring system. In addition, due to financial and resource limitations, we conducted preliminary *in vitro* experiments to investigate the functionality of KYNU in LUAD. The further experimentation and investigation are required for a more complete comprehension of the molecular mechanisms. This study focuses on bioinformatics analysis and preliminary functional investigations to identify possible biomarkers. These limitations will be addressed in future research.

## Conclusions

In conclusion, this study identified disulfidptosis-related subgroups and developed a DS for evaluating the prognosis, immune infiltration, mutations, and treatment sensitivity of LUAD patients. Studying disparities in disulfidptosis patterns has deepened our comprehension of both tumor heterogeneity and the intricate complexities within the TME. We have also constructed and validated a DS that accurately predicts patient prognosis and treatment efficacy assessment, offering a potentially powerful new tool for clinical decision-making, patient outcomes, and individualized treatment strategies. Besides, this study has contributed to advancing the understanding of molecular complexity in LUAD and provides directions and potential avenues for future LUAD research.

## Data availability statement

The datasets presented in this study can be found in online repositories. The names of the repository/repositories and accession number(s) can be found in the article/**Supplementary Material**.

## Ethics statement

The studies involving humans were approved by Ethics Committee and Institutional Review Board of the Outdo Biotech. Co., Ltd. (SHYJS-CP-2210035). The studies were conducted in accordance with the local legislation and institutional requirements. The participants provided their written informed consent to participate in this study.

## Author contributions

CY and YZ conceived and designed the study. CL and YW performed the collection and assembly of data. YX and CL analyzed the data. YZ and YW performed experiments and wrote the manuscript. All authors read and approved the final manuscript.
