# Supplementary material for: Identification of disulfidptosis-related subgroups and prognostic signatures in lung adenocarcinoma using machine learning and experimental validation
Source: Front Immunol. 2023 Sep 20;14:1233260. doi: 10.3389/fimmu.2023.1233260 (PMC10548142; doi:10.3389/fimmu.2023.1233260)
Supplement: Supplementary file 1 [file DataSheet_1.pdf]

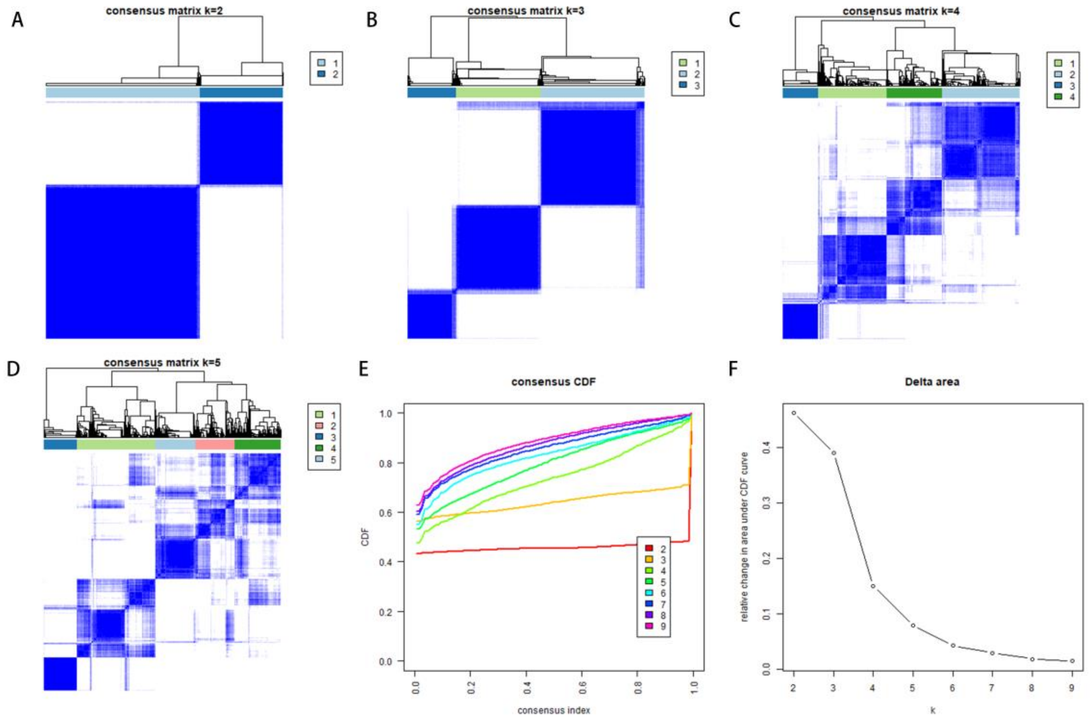


**Supplementary Figure 1** Consensus matrixes of all LUAD patients based on DRs. (A-D) Consensus matrixes of all LUAD patients for each k (k = 2–5). (E) Cumulative distribution function curves for unsupervised clustering of LUAD based on FARGs, k = 2-9. (F) Relative change in area under the CDF curve for unsupervised clustering of LUAD, k =2-9.

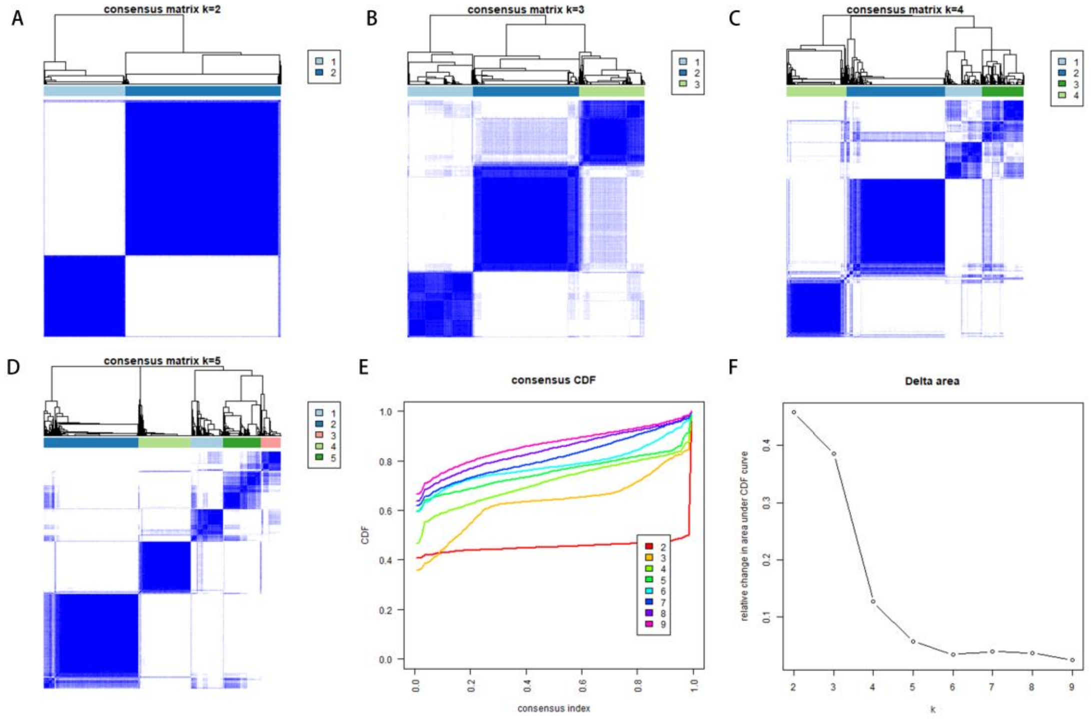


**Supplementary Figure 2** Consensus matrixes of all LUAD patients based on prognostic DEGs. (A-D) Consensus matrixes of all LUAD patients for each k (k = 2–

5). (E) Cumulative distribution function curves for unsupervised clustering of LUAD based on prognostic DEGs, k = 2-9. (F) Relative change in area under the CDF curve for unsupervised clustering of LUAD, k =2-9.

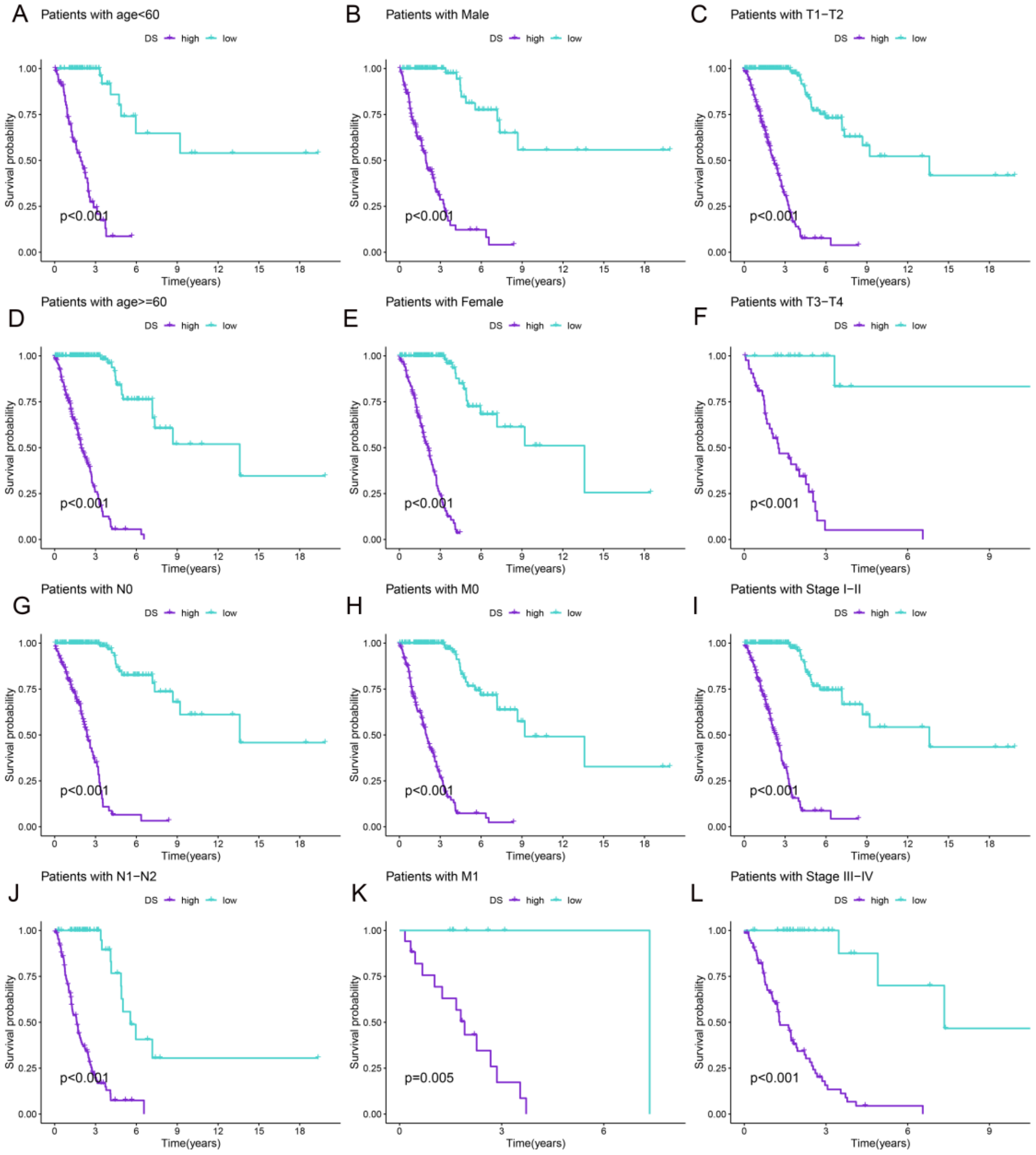


**Supplementary Figure 3** The survival curves of the DS stratified by age, gender, T, N, M and stage. (A) <60 years, (B) male, (C) T1-2, (D) ≥60 years, (E) female, (F) T3-4, (G) N0, (H) M0, (I) stage1-2, (J) N1-2, (K) M1, (L) stage3-4.
