# Supplementary material for: Identification of disulfidptosis-related subgroups and prognostic signatures in lung adenocarcinoma using machine learning and experimental validation
Source: Front Immunol. 2023 Sep 20;14:1233260. doi: 10.3389/fimmu.2023.1233260 (PMC10548142; doi:10.3389/fimmu.2023.1233260)
Supplement: Supplementary file 2 [file DataSheet_2.pdf]

Supplementary Methods

Generation of DS

To establish a robust and accurate consensus model (DS), we employed a comprehensive ensemble of 10 distinct machine learning algorithms. These included the random survival forest (RSF), elastic network (Enet), Lasso, Ridge, stepwise Cox, CoxBoost, partial least squares regression for Cox (plsRcox), supervised principal components (SuperPC), generalized boosted regression modeling (GBM), and survival support vector machine (survival-SVM). Several algorithms demonstrated adeptness in feature selection, including Lasso, stepwise Cox, CoxBoost, and RSF. To create a consolidated model, these algorithms were amalgamated. The amalgamation process involved executing 101 unique algorithm combinations, each tailored to form prediction models within the leave-one-out cross-validation (LOOCV) framework. The inaugural discovery of the signature was performed utilizing TCGA-LUAD data. For the RSF model, we employed the randomForestSRC package, configuring its ntree and mtry parameters through grid-search within the LOOCV framework. The optimal parameter pair (ntree, mtry) resulting in the highest C-index value was selected. Enet, Lasso, and Ridge implementations utilized the glmnet package, determining the regularization parameter λ via LOOCV, while α, the L1-L2 trade-off parameter, was systematically varied from 0 to 1 in intervals of 0.1. Implementing the stepwise Cox model through the survival package involved applying a stepwise algorithm based on the Akaike information criterion (AIC). We experimented with "both", "backward", and "forward" directions for stepwise search. The CoxBoost model was realized using the CoxBoost package, with optimal penalty determined by LOOCV's optimCoxBoostPenalty function. Subsequently, the number of boosting steps was selected via cv.CoxBoost, and the multivariate Cox model's dimension was established by the main CoxBoost routine. For the plsRcox model, the plsRcox package was utilized. We determined the necessary number of components using the cv.plsRcox function, fitting a partial least squares regression generalized linear model with the plsRcox function. The SuperPC model, sourced from the superpc package, extended principal component analysis to extract prominent variation directions within a dataset.

The GBM model was executed through the superpc package. Employing the LOOCV technique, the cv.gbm function efficiently determined the index for the number of trees, aiming to minimize the cross-validation error. Subsequently, the gbm function was applied to effectively configure the generalized boosted regression model. Additionally, the survival-SVM model was established utilizing the survivalsvm package. This model employed a regression approach that thoughtfully incorporated the consideration of censoring. This integration ensured the formulation of inequality constraints for the support vector problem adequately accounted for the specific challenges posed by censoring.
